# AI-Driven Dental Procedure Coding: A Multi-Model Framework for CDT Extraction from Clinical Text

**DOI:** 10.3390/dj14060339

**Published:** 2026-06-02

**Authors:** Pranav Annareddy, Ali Noori, Deepthi Kollipara, Prashanti Manda

**Affiliations:** 1 Department of Computer Science, University of Nebraska Omaha, Omaha, NE 68182, USA; pannareddy@unomaha.edu; 2 Informatics and Analytics, University of North Carolina Greensboro, Greensboro, NC 27412, USA; a_noori@uncg.edu; 3 Summit Dental, Omaha, NE 68136, USA; deepthikollipara88@gmail.com

**Keywords:** dental informatics, automated procedure coding, Large Language Models (LLMs), Clinical Natural Language Processing (NLP)

## Abstract

**Background and Objectives**: Dental procedure coding is essential for accurate billing, reimbursement, and clinical documentation, yet it remains largely manual, time-consuming, and error-prone. While natural language processing (NLP) has enabled significant advances in automated medical coding, limited work has focused on the dental domain, particularly the assignment of Code on Dental Procedures and Nomenclature (CDT) codes from free-text clinical notes. This study aims to develop and evaluate an artificial intelligence framework that integrates large language models (LLMs) and traditional deep learning methods to automate CDT code extraction from narrative dental documentation. **Methods**: We evaluated three LLM-based strategies—zero-shot prompting, QLoRA fine-tuning, and parameter-efficient fine-tuning (PEFT) using LoRA—alongside a supervised Bidirectional GRU (Bi-GRU) classifier. Experiments were conducted using a synthetic dataset designed to emulate real-world dental encounters. Structured JSON output schemas, few-shot prompting, and scalable batch inference pipelines were employed to ensure consistent and interpretable predictions. Model performance was assessed using micro- and macro-averaged F1 scores, precision, recall, exact-match accuracy, and Hamming loss. **Results**: The zero-shot LLM achieved the highest micro-F1 score (0.9614) and perfect recall for frequent CDT codes, demonstrating strong baseline reasoning without task-specific training; however, performance declined for rare procedures and diagnostic code hallucinations were common. Fine-tuning improved domain alignment, with the non-quantized PEFT LoRA model outperforming QLoRA across all metrics, though both fine-tuned LLMs showed tendencies to over-generate plausible but incorrect codes. The Bi-GRU model achieved balanced performance (micro-F1 = 0.9362, macro-F1 = 0.9377) with minimal hallucinations but occasionally missed context-dependent procedures. **Conclusions**: These findings highlight complementary strengths between LLM-based and supervised approaches. LLMs provide strong contextual understanding and rapid deployment, while traditional models offer stable and precise multi-label classification. This work supports the development of hybrid, schema-constrained systems for scalable dental procedure coding.

## 1. Introduction

Dental procedure coding is essential for the clinical and administrative functioning of dental practices. Each patient encounter must be translated into standardized Code on Dental Procedures and Nomenclature (CDT) codes to ensure accurate billing, regulatory compliance, and coordination with insurers [1]. However, this process is often manual, time-consuming, and prone to error. Dental staff must interpret narrative clinical notes and select appropriate CDT codes, a workflow associated with claim denials, financial loss, and reimbursement delays [2]. As healthcare systems increasingly adopt digital infrastructure and value-based care models, there is growing demand for more efficient and reliable dental coding solutions [3].

Artificial intelligence (AI) has emerged as a major technological driver in modern dentistry. Across specialties such as orthodontics, AI methods are now widely studied and applied to tasks including landmark detection, growth prediction, and treatment planning [4]. More broadly, machine learning and deep learning systems are increasingly used to support diagnostic imaging, predictive analytics, and clinical decision-making. This growing adoption of AI in dentistry motivates exploration of its use beyond imaging—particularly in structured information extraction and automated coding from clinical documentation.

In medical informatics, automated coding from clinical text has advanced significantly due to developments in Natural Language Processing (NLP). Early approaches relied on traditional machine learning models such as logistic regression and support vector machines [5], followed by recurrent neural networks capable of modeling sequential text patterns [6]. More recently, transformer-based models—including BERT and GPT architectures—have demonstrated strong performance in extracting structured codes from complex clinical narratives [7,8]. These advances show that AI systems can effectively map unstructured text to standardized coding schemes.

Despite progress in medical NLP, the dental domain remains comparatively underexplored. A systematic review found that dental NLP research is limited in scope and methodological consistency, with few studies addressing structured coding tasks [9]. Compared to ICD or SNOMED Clinical Terminology (CT) coding in medicine, CDT-based procedure coding has received little attention in AI research. Dental clinical notes present unique challenges: procedures are often described implicitly, terminology varies among providers, and multiple procedures may occur in a single encounter [10]. These characteristics make automated CDT coding both challenging and clinically relevant.

Large Language Models (LLMs) offer new opportunities for addressing this problem. LLMs can perform few-shot learning, allowing them to infer coding patterns from limited examples [11]. They can also generate structured outputs, such as JSON-formatted responses, that integrate directly into billing and electronic health record systems [12]. At the same time, traditional neural architectures such as Bidirectional GRU models provide efficient supervised baselines for structured multi-label classification [13].

This study presents an end-to-end AI framework for automated CDT coding from free-text dental clinical notes. The system integrates zero-shot and fine-tuned LLM approaches with a supervised Bi–GRU classifier and evaluates performance using micro- and macro-averaged F1 scores, precision, recall, exact-match accuracy, and Hamming loss. The goal is to assess the feasibility, strengths, and limitations of generative and discriminative AI methods for dental procedure coding.

To our knowledge, this is one of the first comprehensive studies to evaluate multiple AI paradigms for multi-label CDT extraction from unstructured dental notes. The key contributions of this work are:

- An end-to-end framework for automated CDT coding from narrative dental documentation.
- A structured JSON-based schema for machine-readable CDT extraction.
- A comparative evaluation of zero-shot LLMs, parameter-efficient fine-tuning methods, and a traditional Bi–GRU classifier.
- A scalable inference pipeline suitable for batch processing and reproducible evaluation.

Together, these contributions provide a technical foundation for AI-driven dental coding and support future development of automated systems aimed at improving efficiency and accuracy in dental documentation and billing [14].

## 2. Related Work

Automated coding from clinical text has been extensively studied in the medical domain, particularly for ICD and procedure code prediction from Electronic Health Record (EHR) narratives. Classical machine learning methods and deep learning architectures—including convolutional neural networks (CNNs), recurrent neural networks (RNNs), and transformer-based models—have demonstrated strong performance in mapping free-text clinical documentation to structured billing and diagnostic codes [6,13,15]. These works established the feasibility of large-scale multi-label classification from clinical text and provided the methodological foundation for applying natural language processing (NLP) to structured coding tasks.

In contrast, dental clinical documentation has received comparatively limited attention within the NLP community. A systematic review by Stohl et al. identified only seventeen studies applying NLP to dental records between 2015 and 2021, of which fewer than two-thirds implemented substantive computational modeling approaches [9]. The review concluded that dental NLP research remains heterogeneous, under-standardized, and constrained by limited annotated datasets, with minimal focus on billing-oriented procedural coding. Similarly, Pethani and Dunn emphasized that while dental notes contain rich clinical information suitable for structured extraction, reproducibility and generalizability remain ongoing challenges due to variability in documentation practices and restricted data availability [9]. Buttner et al. further highlighted both the opportunities and limitations of applying modern transformer-based NLP methods in dentistry, noting that while deep learning approaches may outperform rule-based systems, their success depends heavily on labeled data volume and domain-specific validation [16].

Recent applied studies illustrate the feasibility of structured information extraction from dental electronic health records when the task is well-defined. Keels et al. developed a periodontal diagnosis extraction system using synthetic data generated via a large language model and a fine-tuned RoBERTa classifier, achieving weighted average F1 scores above 0.97 across diagnostic categories [10]. Similarly, Chuang et al. explored periodontal entity extraction workflows that leverage LLM-based prompt generation to create seed examples for downstream RoBERTa-based named entity recognition models, demonstrating strong extraction performance and highlighting the potential of LLM-assisted data curation strategies [17]. Follow-up work by Chuang et al. further emphasized the importance of cross-institutional validation for dental NLP systems to ensure robustness across heterogeneous documentation environments [18].

More broadly, reviews by Ding et al. and Eggmann et al. discuss the expanding role of artificial intelligence in dentistry, including opportunities for workflow automation, patient communication enhancement, and insurance processing support [14,19]. Early proof-of-concept studies in specialty areas such as endodontics also demonstrate the feasibility of NLP-assisted charting and documentation automation [20,21]. Collectively, these works signal growing interest in applying AI to dental documentation and administrative processes.

Despite this momentum, important gaps remain. Existing dental NLP studies primarily focus on diagnostic entity extraction, periodontal staging, or narrow classification tasks rather than end-to-end procedure coding aligned with billing standards. To our knowledge, no prior published work has implemented a comprehensive system for multi-label CDT (Code on Dental Procedures and Nomenclature) extraction directly from unstructured dental provider notes. Furthermore, prior studies rarely incorporate structured, schema-constrained outputs suitable for integration into electronic dental record (EDR) systems, and few compare zero-shot LLM inference, parameter-efficient fine-tuning, and traditional supervised neural architectures within a unified procedural coding framework.

This study addresses these gaps by introducing a comprehensive multi-model framework for automated CDT code extraction from free-text dental clinical notes. By integrating zero-shot large language models, parameter-efficient fine-tuned LLMs, and a traditional Bi–GRU classifier within a structured JSON output schema designed for billing workflow compatibility, this work advances the methodological rigor and practical applicability of dental coding automation research.

## 3. Methods

### 3.1. Dataset

The dataset used in this study consists of a synthetic corpus of dental clinical encounters designed to emulate the structure, variability, and linguistic characteristics of real-world dental documentation. The dataset is stored in a structured CSV format and contains one row per dental visit, with each record representing a unique clinical note accompanied by metadata and ground-truth coding information. Although synthetic, the data was generated to reflect realistic clinical scenarios, incorporating common dental conditions, procedural descriptions, patient demographics, and annotation formats typically observed in dental practice management systems.

Each record includes a unique note identifier (note_id), encounter date, patient demographic attributes (sex and age), and optional contextual information such as risk factors (e.g., xerostomia, poor oral hygiene, high-sugar diet) and allergies (e.g., penicillin, latex). The core of the dataset is the note_text field, which contains richly detailed narrative clinical notes formatted in a style consistent with dentist-authored documentation. These notes describe presenting symptoms, diagnosed conditions, intraoral findings, tooth-specific observations, and treatment plans.

To support supervised modeling and evaluation, each clinical note is paired with two structured annotation fields. The gold_annotations column contains fine-grained, mention-level annotations indicating clinically relevant entities within the note—such as “mesial caries on tooth #14” or “irreversible pulpitis suspected on tooth #3”—together with structured metadata including tooth number, surface, and condition. The suggested_procedures field provides the corresponding gold-standard CDT procedure codes and descriptive terms associated with each encounter (e.g., CDT-D2140 for amalgam restoration, CDT-D3310 for root canal therapy on anterior teeth, CDT-D2740 for porcelain crowns). These fields serve as the supervised targets for model training and evaluation.

Overall, the dataset mirrors the complexities required for realistic dental coding tasks: multi-label code assignments, heterogeneous provider language, tooth-level specificity, and clinically meaningful variation across notes. Its structured design enables experimentation with instruction-tuned LLMs, traditional deep learning models, and benchmarking of inference pipelines under controlled, reproducible conditions.

### 3.2. Synthetic Clinical Note Generation

Because access to real electronic dental record (EDR) data was not available for this study, we constructed a synthetic dataset of dental clinical notes designed to simulate common documentation patterns observed in outpatient dental practice. The goal of this dataset was to create controlled yet clinically plausible narrative descriptions of dental encounters that could be used to evaluate automated CDT code extraction.

Synthetic notes were generated using a combination of predefined clinical templates and variable text components. Each template corresponded to a common dental procedure category (e.g., preventive examination, prophylaxis, tooth extraction, restorative procedures). Within each template, variable elements such as tooth number, procedure modifiers, clinical findings, anesthesia details, and post-operative instructions were randomly instantiated to introduce variation across notes.

For each generated note, the corresponding CDT code labels were assigned based on the procedures described in the scenario. Multiple procedures could be included in a single note, allowing the task to be formulated as a multi-label classification problem. This design reflects real dental encounters where several procedures may be documented during a single visit.

To increase linguistic diversity, multiple paraphrased templates were created for each procedure type. These templates varied in sentence structure, terminology, and level of detail while preserving the same underlying clinical meaning. Random combinations of these templates were used to produce narrative notes with differing phrasing and ordering of information.

Examples of simulated clinical scenarios included routine preventive visits, restorative procedures involving composite fillings, simple tooth extractions, and encounters involving multiple procedures performed during the same appointment. Each generated note contained free-text documentation resembling typical provider-authored clinical narratives rather than structured fields.

An example of a synthetic preventive visit note is shown below:

*“Patient presented for routine dental examination and cleaning. Comprehensive oral evaluation performed. No acute pathology observed. Prophylaxis completed using ultrasonic scaler and hand instruments. Oral hygiene instructions provided. Patient tolerated the procedure well.”*

An example restorative scenario:

*“Patient reported sensitivity on tooth #19. Examination revealed occlusal caries. Local anesthesia administered. Caries removed and composite restoration placed. Occlusion checked and adjusted. Post-operative instructions discussed.”*

An example extraction scenario:

*“Patient presented with non-restorable tooth #30 due to extensive decay. Local anesthesia achieved using lidocaine. Simple extraction performed with elevator and forceps. Hemostasis achieved and post-extraction instructions provided.”*

By combining multiple templates with variable clinical elements, the dataset produced a diverse set of notes representing common dental encounters while maintaining deterministic ground-truth CDT labels for evaluation.

### 3.3. Analysis Plan

This study employed a comprehensive multi-method artificial intelligence pipeline to automate the extraction of CDT procedure codes from free-text dental clinical notes. The methodology integrates both state-of-the-art Large Language Models (LLMs) and traditional deep learning architectures to evaluate their performance on a complex multi-label classification task. The workflow includes the construction of a structured output schema and few-shot learning examples, advanced prompt engineering for controlled LLM inference, and a scalable batch-processing system for large dataset evaluation. We implemented a full evaluation framework using micro- and macro-level F1 metrics and Hamming loss to assess predictive accuracy across common and rare dental procedures. In addition, we developed a complete fine-tuning pipeline using parameter-efficient QLoRA techniques to adapt a pretrained TinyLlama model to domain-specific dental coding tasks, followed by tokenization, model initialization, and inference deployment. To provide a comparative baseline, we also trained and evaluated a Bidirectional GRU neural network using a standardized text preprocessing and multi-label binarization approach. Together, these methods enable a rigorous assessment of LLM-based reasoning versus traditional neural architectures and provide a robust foundation for developing deployable dental coding automation tools.

#### 3.3.1. LLM-Based Dental Coding

This study develops a comprehensive Large Language Model (LLM)-driven framework for the automated extraction of CDT procedure codes from free-text dental clinical notes. The system leverages structured schema design, prompt-based learning, fine-tuning strategies, and both generative and discriminative neural architectures to enable accurate, consistent, and interpretable prediction of dental procedure codes. The sections below describe each component of the LLM-based pipeline and the corresponding modeling approaches.

- Data Establishment and Structured Output Schema

The LLM-based system begins with the creation of a carefully curated set of example dental notes and a structured output format. A few-shot learning strategy is implemented using the FEW_SHOT_EXAMPLES variable, which contains realistic clinical note excerpts paired with their corresponding CDT procedure codes. These examples expose the LLM to patterns in dental documentation, enabling it to infer mappings between narrative descriptions and standardized codes without explicit rule-based programming.

To ensure consistent and machine-readable outputs, the LLM_PROMPT_SCHEMA defines a strict JSON-based response template. Predictions must adhere to this structure, including an array of CDT code strings and optional evidence items that reference supporting text spans. This schema supports downstream integration into billing workflows, auditing systems, and analytic dashboards.

- Prompt Engineering and LLM Inference System

Advanced prompt engineering techniques are used to maximize LLM performance for zero-shot and few-shot inference. For each dental note, a dynamically constructed prompt incorporates selected few-shot examples, explicit task instructions, and the structured JSON output schema. The prompt explicitly frames the model as a “dental coding assistant,” which significantly improves the reliability of generated outputs.

A dedicated single-note inference function assembles the prompt, sends it to the OpenAI API using deterministic decoding parameters (temperature = 0), and extracts valid JSON objects via regular expressions. This enables real-time and interactive usage in clinical settings where automated CDT code suggestions may be required at the point of care.

- Large-Scale Batch Inference Pipeline

To support production-scale experimentation, the framework includes a batch inference pipeline capable of processing large collections of notes efficiently. A randomized subset of up to 100 records is selected for evaluation with reproducibility ensured via fixed random states. Records are processed in batches of 50 to balance throughput and API usage costs.

After each batch is completed, predictions are immediately written to a persistent JSONL file. This enables checkpointing, fault tolerance, and recovery in the event of interruptions. Each entry contains the original note, gold-standard CDT codes, and the model-predicted codes, supporting downstream evaluation and error analysis.

- Modeling Approaches

We implemented and compared four distinct modeling approaches, each differing in training requirements, inference mechanisms, and evaluation strategies (Table 1).

- Low-/Zero-Shot LLM (GPT-4o-mini): This approach requires no model training and relies entirely on prompt-based inference, making it the most lightweight and immediately deployable strategy for automated dental coding. In the *zero-shot* setting, the model receives only the raw clinical note together with high-level task instructions (e.g., “extract CDT procedure codes from this note and return them in JSON format”), without being shown any explicit examples. Zero-shot prompting leverages the LLM’s broad world knowledge and inherent reasoning capabilities to interpret dental terminology, infer the procedures described in the text, and map them to the appropriate CDT codes. In the *low-shot* or *few-shot* setting, the model is additionally provided with a small number of example clinical notes paired with their correct CDT codes. These demonstrations guide the model toward the desired transformation by illustrating how similar inputs should be converted into structured outputs. When clinical notes are passed to the LLM along with these curated few-shot examples and the JSON output schema, the model generates CDT predictions entirely through inference rather than parameter updates. Because no fine-tuning occurs, no train/test split is required for this method.
- Fine-tuned LLMs: Fine-tuning Large Language Models (LLMs) represents a powerful strategy for adapting general-purpose models to specialized domains such as dental informatics. Although state-of-the-art LLMs demonstrate strong general reasoning and language understanding, their zero-shot performance may be limited when applied to highly domain-specific terminology, coding conventions, and contextual nuances found in clinical narratives. Fine-tuning enables a model to internalize these patterns by training on curated examples of dental notes paired with correct CDT codes, thereby improving predictive accuracy, reducing hallucinations, and ensuring greater conformity to structured output schemas required in billing workflows. Parameter-efficient fine-tuning approaches such as LoRA and QLoRA further enhance practicality by updating only a small subset of model parameters rather than the entire network, enabling domain adaptation on modest hardware without sacrificing performance. For these reasons, fine-tuning constitutes a critical avenue for building robust, domain-specialized dental coding systems.
  1. QLoRA Fine-Tuning (TinyLlama 1.1B): A 4-bit quantized TinyLlama model is fine-tuned using the QLoRA (Quantized Low-Rank Adaptation) framework, which enables efficient domain adaptation on consumer hardware without compromising model performance. QLoRA loads the pretrained model weights in 4-bit NormalFloat (NF4) quantization, greatly reducing memory requirements while maintaining high representational fidelity. Rather than updating the full set of model parameters, QLoRA injects small low-rank adapter matrices into targeted transformer layers, and only these adapters are trained. The underlying quantized backbone remains frozen, retaining the model’s general linguistic knowledge. This parameter-efficient strategy makes it feasible to fine-tune a billion-parameter LLM such as TinyLlama on modest GPU resources.
    The dental dataset is divided into a 90% training and 10% testing split. Each clinical note is converted into an instruction-style prompt conforming to the JSON output schema, and the associated CDT codes serve as supervised labels. Fine-tuning proceeds with minimal memory overhead due to quantization-aware optimization, gradient checkpointing, and the small number of trainable parameters in the LoRA adapters.
    After fine-tuning, inference is conducted via a HuggingFace text-generation pipeline configured with half-precision weights and automatic device placement. For each new clinical note, the model generates JSON-formatted CDT predictions, which are parsed to extract the predicted codes. Evaluation uses the same metrics applied to the zero-shot model—micro- and macro-averaged F1, precision, recall, exact-match accuracy, and Hamming loss—facilitating direct comparison between prompt-only and fine-tuned performance.
  2. PEFT LoRA Fine-Tuning: A second fine-tuning strategy employs Parameter-Efficient Fine-Tuning (PEFT) through LoRA (Low-Rank Adaptation) adapters without applying 4-bit quantization to the base model. In this configuration, the pretrained TinyLlama model is loaded in full or half precision, preserving the full representational capacity of the underlying transformer weights. LoRA inserts lightweight trainable matrices into targeted components of the model—such as the query, key, value, and output projection layers—and only these adapter parameters are updated during training, while the original model weights remain frozen. This approach substantially reduces the number of trainable parameters while enabling the model to learn domain-specific patterns in dental clinical text.
    By avoiding quantization, the LoRA-only configuration maintains higher numerical precision, which may be advantageous when modeling rare or nuanced linguistic cues present in clinical narratives. As in the QLoRA setup, the dataset is divided into a 90% training and 10% testing split. Instruction-style prompts paired with gold-standard CDT labels provide the supervised training signal, allowing the model to learn the structured mapping from narrative input to multi-label output.
    Following fine-tuning, inference is conducted using a HuggingFace text-generation pipeline, with the LoRA-enhanced model producing JSON-formatted CDT code predictions. These outputs are parsed and compared with human-annotated labels. Evaluation uses micro- and macro-averaged F1, precision, recall, exact-match accuracy, and Hamming loss, matching the metrics applied to other modeling strategies.
    Importantly, this LoRA configuration functions as a quantization-free baseline for assessing the impact of 4-bit compression in QLoRA. By isolating the quantization variable, the comparison between LoRA and QLoRA enables a clear understanding of the trade-offs between computational efficiency and predictive accuracy when deploying LLM-based dental coding systems.
    For clarity, different base models were used for the two parameter-efficient fine-tuning configurations. The QLoRA experiments were conducted using the TinyLlama-1.1B-Chat-v1.0 model due to its compatibility with 4-bit quantization workflows. In contrast, the PEFT (LoRA) experiments were performed using the Phi-1.5 model without quantization to provide a full-precision baseline for comparison.

#### 3.3.2. Bi-GRU Neural Network

A traditional neural architecture is also implemented for comparison through a Bidirectional Gated Recurrent Unit (Bi–GRU) network, which serves as a strong baseline for sequence modeling tasks in clinical text classification. Prior to modeling, all input dental notes are lowercased, tokenized using a Keras tokenizer with a fixed vocabulary size of 10,000 words, and converted into integer sequences. These sequences are then padded or truncated to a uniform length of 100 tokens to ensure consistent input dimensionality for batch processing. The model begins with an embedding layer that maps each token to a 128-dimensional dense vector, enabling the network to learn semantic and contextual representations of dental terminology. The embedded sequences are subsequently passed through a bidirectional GRU layer with 64 units in each direction, allowing the model to capture both forward and backward contextual dependencies in the narrative clinical notes.

The final hidden representation is routed through dropout layers to mitigate overfitting, followed by a fully connected sigmoid-activated output layer that produces a probability for each CDT code in the multi-label classification space. During inference, these probabilities are thresholded at 0.5 to determine which codes are predicted for each note. The dataset is split into 80% training and 20% testing, with 10% of the training portion held out for validation. Model optimization is performed using binary cross-entropy loss and the Adam optimizer. Performance is evaluated using micro- and macro-averaged F1 scores, Hamming loss, and per-label performance reports, enabling assessment of both common and rare dental procedure codes. This Bi–GRU model provides a strong traditional baseline against which fine-tuned LLM-based approaches can be compared.

### 3.4. Evaluation Framework

All models—zero-shot LLMs, fine-tuned LLMs, and the Bi–GRU neural network—are evaluated against human-annotated CDT labels using a comprehensive suite of multi-label classification metrics (Table 1). Micro- and macro-averaged F1 scores are used to assess the balance between precision and recall. The *micro-F1* score aggregates contributions from all labels and therefore emphasizes performance on more frequent CDT codes, making it especially informative in imbalanced datasets. In contrast, the *macro-F1* score computes F1 values independently for each CDT code and then averages them, treating common and rare procedures equally and highlighting the model’s ability to generalize across the full label space.

Precision and recall provide complementary insights into prediction quality: precision quantifies the proportion of predicted codes that are correct, while recall reflects the proportion of true codes successfully retrieved. These metrics are critical in dental billing contexts, where false positives may inflate claims and false negatives may omit billable procedures. To assess holistic coding accuracy, *exact-match accuracy* is calculated as the proportion of clinical notes for which the model predicts the entire set of CDT codes exactly. This stringent metric evaluates the extent to which a model can fully automate the multi-label coding task. Additionally, *Hamming loss* measures the fraction of incorrectly predicted labels across all label decisions, accounting for both false positives and false negatives at the code level. This metric is particularly useful for characterizing partial prediction errors in multi-label settings. Together, these metrics provide a multidimensional evaluation of model performance, capturing global predictive behavior, fine-grained code-level accuracy, and strict end-to-end coding fidelity.

### 3.5. Fine-Tuning Hyperparameter Configuration

To ensure reproducibility and transparency of the fine-tuning experiments, the hyperparameter configurations for both QLoRA and standard LoRA models are reported here (Table 2).

The QLoRA experiments were conducted using the TinyLlama-1.1B-Chat-v1.0 model as the base architecture. Model weights were loaded using 4-bit NormalFloat (NF4) quantization with bfloat16 computation and double quantization enabled to reduce memory usage while preserving representational fidelity. Low-Rank Adaptation (LoRA) adapters were applied to the transformer projection layers with rank r=64, scaling parameter α=16, and a dropout rate of 0.05. The target modules included the attention projection layers (qkv_proj) and output projection layer (o_proj). Training was performed for one epoch using a learning rate of $2\times 10^{-4}$. The per-device batch size was set to 2 with gradient accumulation steps of 4, resulting in an effective batch size of 8. Mixed-precision training (FP16) was enabled, and logging was performed every 200 training steps.

For the non-quantized parameter-efficient fine-tuning (PEFT) LoRA configuration, the TinyLlama model was loaded without low-bit quantization. LoRA adapters were applied to the query, value, and output projection layers with rank r=8, α=16, and dropout rate of 0.05. Training was again performed for one epoch using a learning rate of $2\times 10^{-4}$, a per-device batch size of 1, and gradient accumulation steps of 4, resulting in an effective batch size of 4.

All experiments used a consistent dataset preparation pipeline. Clinical notes were truncated or padded to a maximum sequence length of 256 tokens. The dataset was randomly partitioned using a fixed seed (42) into 90% training data and 10% testing data.

During training, each clinical note was converted into an instruction-style prompt that asked the model to return CDT procedure codes in JSON format.

These hyperparameters were selected to balance training stability, computational feasibility, and memory efficiency on available hardware while enabling direct comparison between quantized and non-quantized parameter-efficient fine-tuning strategies.

#### Prompt Template and Few-Shot Examples

For LLM-based inference, clinical notes were converted into instruction-style prompts designed to guide the model toward structured CDT code extraction. The prompt instructs the model to act as a dental coding assistant and return predicted procedure codes in JSON format. This structure ensures consistent outputs that can be automatically parsed during evaluation.

A simplified version of the prompt template is shown below:

You are a dental coding assistant.

Note: {clinical_note}

Return CDT procedure codes in JSON format.

Output:

Few-shot examples were included in the prompt to illustrate the mapping between narrative clinical documentation and standardized CDT codes. These examples help the model learn the expected output format and coding behavior.

Few-Shot Example 1

Input:

Caries removal and composite restoration on tooth #12.

Output:

{“codes”: [“D2330”]}

Few-Shot Example 2

Input:

Root canal therapy performed on molar due to irreversible pulpitis.

Output:

{“codes”: [“D3330”]}

Few-Shot Example 3

Input:

Patient presented with non-restorable tooth #30. Local anesthesia administered and simple extraction performed.

Output:

{“codes”: [“D7140”]}

During inference, each clinical note was inserted into the prompt template together with the few-shot examples, and the LLM generated CDT code predictions using deterministic decoding (temperature = 0). The resulting JSON outputs were parsed to extract the predicted CDT codes for downstream evaluation.

## 4. Results

The final dataset contained 10 unique CDT procedure codes with varying frequencies across clinical scenarios (Table 3). The distribution was moderately imbalanced, with several procedures occurring much more frequently than others. The most common procedure was CDT-D4341 (periodontal scaling and root planing), which appeared 3530 times, followed by CDT-D2740 (porcelain crown) with 2213 instances and CDT-D1110 (adult prophylaxis) with 1938 instances. Other commonly represented procedures included CDT-D7140 (extraction of an erupted tooth) and CDT-D3330 (molar root canal therapy). Several restorative and endodontic procedures (e.g., CDT-D3310, CDT-D3320, and CDT-D2392) were present at moderate frequencies.

In contrast, CDT-D6010 (dental implant placement) appeared only 71 times, representing the rarest procedure in the dataset. This variation in procedure frequency reflects the diversity of simulated clinical encounters and introduces a degree of class imbalance typical of multi-label clinical coding tasks. Such imbalance provides a realistic testing scenario for evaluating the robustness of automated coding models across both common and infrequent procedures.

### 4.1. Performance of LLM-Based Approaches

The results show notable differences in performance across the three modeling strategies. The low/zero-shot model achieves the highest micro-F1 score (0.9614) and perfect micro-recall, indicating exceptionally strong performance on the most frequent CDT codes—even without training (Table 4). However, its macro-F1 score (0.4543) suggests reduced ability to capture rare or less frequently seen procedures, which is expected given that zero-shot prompting relies on general knowledge rather than domain adaptation. In contrast, the QLoRA fine-tuned model performs substantially worse across all metrics, particularly in exact match (0.39) and macro-F1 (0.2165), highlighting that aggressive 4-bit quantization combined with limited domain examples may hinder the model’s ability to generalize across the full label set. The standard LoRA fine-tuning approach (PEFT without quantization) performs considerably better than QLoRA, achieving strong micro-F1 (0.9136) and exact-match accuracy (0.79), and improves substantially on macro-level metrics, suggesting better handling of rare labels. The higher number of predicted unique labels in QLoRA (42 vs. 27) indicates a tendency toward over-prediction, which contributes to higher Hamming loss. Overall, these results demonstrate that while zero-shot prompting remains highly effective for frequent CDT codes, parameter-efficient fine-tuning—particularly non-quantized LoRA—offers meaningful gains in structured prediction accuracy and improved robustness across diverse procedure categories.

### 4.2. Bi-GRU Performance

The Bi-GRU model demonstrates strong and highly balanced performance across both micro- and macro-level metrics, with micro_f1 = 0.9362 and macro_f1 = 0.9377 (Table 5). This near-equivalence between micro and macro scores indicates that the model performs consistently well across both frequent and rare CDT labels—an outcome that contrasts with LLM-based approaches, which often show a substantial gap between micro- and macro-level performance. The relatively low Hamming loss (0.0185) further suggests that the Bi-GRU makes few label-level errors and maintains high precision in multi-label predictions. Notably, the macro-F1 score exceeding 0.93 reflects the model’s ability to generalize effectively across diverse procedures, including those underrepresented in the dataset. These results highlight that a traditional neural sequence model, when trained directly on domain-specific data, can deliver robust and reliable performance, serving as a strong baseline and, in some cases, outperforming fine-tuned LLMs on structured multi-label classification.

### 4.3. Comparison of LLM vs. Bi-GRU Approaches

Compared with the LLM-based methods, the Bi-GRU model demonstrates a markedly different performance profile that highlights the strengths and trade-offs of traditional neural architectures versus large generative models. The Bi-GRU achieves micro- and macro-F1 scores above 0.93, indicating uniformly strong performance across both frequent and rare CDT codes. This contrasts with the zero-shot and fine-tuned LLMs, which exhibit a substantial disparity between micro- and macro-level metrics, reflecting their tendency to excel on common procedures while struggling with underrepresented or nuanced codes. The balanced precision and recall of the Bi-GRU suggest that direct supervised learning on domain-specific data enables the model to internalize fine-grained patterns that LLMs—especially prompt-only or lightly fine-tuned models—may overlook. However, while the Bi-GRU offers strong structured prediction capabilities, it lacks the broader contextual reasoning ability of LLMs and depends heavily on labeled datasets for training. In contrast, LLMs provide flexibility, adaptability, and strong zero-shot performance without requiring annotated data, making them attractive for rapid deployment. Ultimately, the comparison highlights a key trade-off: Bi-GRU models excel when high-quality domain-specific training data is available, whereas LLMs offer greater generalization and ease of use, but may require careful fine-tuning to achieve comparable multi-label accuracy.

### 4.4. Error Analysis

To better understand how each model behaves on real-world SNODENT–CDT prediction tasks, we conducted a qualitative error analysis. For every model (Zero-Shot LLM, QLoRA fine-tuned LLM, PEFT LLM, and Bi-GRU), we provide representative examples of correctly predicted and incorrectly predicted examples. Each example includes the full sentence, expected CDT codes, and model-predicted codes.

#### 4.4.1. Zero-Shot Model

##### Correctly Predicted Samples

- Correct Sample 1 Sentence: “Date: 2024-04-24 …Procedures: CDT-D7140 Extraction.” Expected: D7140; Predicted: D7140.
- Correct Sample 2 Sentence: “Date: 2022-02-23 …Procedures: CDT-D3330 Root canal therapy; CDT-D4341 Scaling and root planing.” Expected: D3330, D4341; Predicted: D3330, D4341.
- Correct Sample 3 Sentence: “Date: 2022-01-21 …Procedures: CDT-D1110 Adult prophylaxis.” Expected: D1110; Predicted: D1110.
- Correct Sample 4 Sentence: “Date: 2024-01-31 …Procedures: CDT-D1110 Adult prophylaxis (cleaning).” Expected: D1110; Predicted: D1110.
- Correct Sample 6 Sentence: “Date: 2024-11-11 …Procedures: CDT-D3320 Root canal therapy; CDT-D2740 Crown.” Expected: D3320, D2740; Predicted: D3320, D2740.
- Correct Sample 7 Sentence: “Date: 2023-10-16 …Procedures: CDT-D4341 Scaling and root planing; CDT-D3330 Root canal therapy.” Expected: D4341, D3330; Predicted: D4341, D3330.
- Correct Sample 8 Sentence: “Date: 2022-07-11 …Procedures: CDT-D3330 Root canal therapy.” Expected: D3330; Predicted: D3330.
- Correct Sample 9 Sentence: “Date: 2024-04-01 …Procedures: CDT-D1110 Adult prophylaxis.” Expected: D1110; Predicted: D1110.
- Correct Sample 10 Sentence: “Date: 2023-03-25 …Procedures: CDT-D4341; CDT-D2740.” Expected: D4341, D2740; Predicted: D4341, D2740.
- Correct Sample 10 (variant) Sentence: “Date: 2023-01-28 …Procedures: CDT-D1110, CDT-D7140, CDT-D2392.” Expected: D1110, D7140, D2392; Predicted: D1110, D7140, D2392.

##### Incorrectly Predicted Samples

- Incorrect Sample Sentence: “Date: 2022-06-11 …Procedures: CDT-D1110 Adult prophylaxis.” Expected: D1110; Predicted: D1110, SNODENT-SND-EXM-000.
- Incorrect Sample Sentence: “Date: 2023-10-22 …Procedures: CDT-D4341 Scaling and root planing.” Expected: D4341; Predicted: D4341, SNODENT-SND-PER-010.
- Incorrect Sample Sentence: “Date: 2025-04-01 …Procedures: CDT-D3320 Root canal therapy.” Expected: D3320; Predicted: D3320, SNODENT-SND-ABS-021.
- Incorrect Sample Sentence: “Date: 2022-02-04 …Procedures: CDT-D4341 Scaling and root planing.” Expected: D4341; Predicted: D4341, SNODENT-SND-PER-010.
- Incorrect Sample Sentence: “Date: 2023-03-20 …Procedures: CDT-D3330 Root canal therapy.” Expected: D3330; Predicted: D3330, SNODENT-SND-PUL-020.

#### 4.4.2. QLoRA Fine-Tuned Model

##### Correctly Predicted Samples

- Correct Sample Sentence: “Date: 2022-02-23 …Procedures: CDT-D3330; CDT-D4341.” Expected: D3330, D4341; Predicted: D4341, D3330.
- Correct Sample Sentence: “Date: 2022-01-21 …Procedures: CDT-D1110.” Expected: D1110; Predicted: D1110.
- Correct Sample Sentence: “Date: 2024-11-11 …Procedures: CDT-D3320; CDT-D2740.” Expected: D3320, D2740; Predicted: D2740, D3320.
- Correct Sample Sentence: “Date: 2023-10-16 …Procedures: CDT-D4341; CDT-D3330.” Expected: D4341, D3330; Predicted: D4341, D3330.

##### Incorrectly Predicted Samples

- Incorrect Sample Sentence: “Date: 2024-04-24 …Procedures: CDT-D7140.” Expected: D7140; Predicted: CDT-K0409, D7140.
- Incorrect Sample Sentence: “Date: 2022-06-11 …Procedures: CDT-D1110.” Expected: D1110; Predicted: D1790, D1110, D1990.
- Incorrect Sample Sentence: “Date: 2022-07-11 …Procedures: CDT-D3330.” Expected: D3330; Predicted: P0310, D1110, D3330.

#### 4.4.3. PEFT (Phi-1.5) Model

##### Correctly Predicted Samples

- Correct Sample Sentence: “Date: 2022-02-23 …Procedures: CDT-D3330; CDT-D4341.” Expected: D3330, D4341; Predicted: D4341, D3330.
- Correct Sample Sentence: “Date: 2024-06-01 …Procedures: CDT-D7140; CDT-D3320; CDT-D2740.” Expected: D7140, D3320, D2740; Predicted: D2740, D7140, D3320.

##### Incorrectly Predicted Samples

- Incorrect Sample Sentence: “Date: 2024-04-24 …Procedures: CDT-D7140.” Expected: D7140; Predicted: CDT-T1234, D7140.
- Incorrect Sample Sentence: “Date: 2022-01-21 …Procedures: CDT-D1110.” Expected: D1110; Predicted: D4341, D1110.

#### 4.4.4. Bi-GRU Model

##### Correctly Predicted Samples

- Correct Sample Sentence: “Date: 2024-04-24 …Procedures: CDT-D7140.” Expected: D7140; Predicted: D7140.
- Correct Sample Sentence: “Date: 2022-02-23 …Procedures: CDT-D3330; CDT-D4341.” Expected: D3330, D4341; Predicted: D3330, D4341.

##### Incorrectly Predicted Samples

- Incorrect Sample Sentence: “Date: 2023-10-16 …Procedures: CDT-D4341; CDT-D3330.” Expected: D3330, D4341; Predicted: D4341.
- Incorrect Sample Sentence: “Date: 2023-01-28 …Procedures: CDT-D1110; CDT-D7140; CDT-D2392.” Expected: D1110, D7140, D2392; Predicted: D1110.

### 4.5. Summary of Common Error Patterns Across Models

A qualitative comparison of model behaviors reveals that each architecture exhibits distinct and recurrent error patterns. These patterns highlight fundamental differences in how neural sequence models and large language models interpret dental clinical narratives, assign CDT procedure codes, and generalize across diagnostic and procedural contexts.

#### 4.5.1. Zero-Shot LLM

The zero-shot model frequently over-generates codes by appending SNODENT diagnostic concepts as if they were CDT procedure codes. This reflects its difficulty in separating the semantic space of diagnoses (SNODENT) from billing procedures (CDT). Although the model identifies relevant context such as periodontal disease or pulpitis, it often treats diagnostic terms as actionable procedures. Errors are dominated by *hallucinated additions*, while omissions of required CDT codes are comparatively rare.

#### 4.5.2. QLoRA Fine-Tuned Model

Fine-tuning substantially improves alignment with CDT coding conventions, but the model still introduces hallucinated CDT codes that are plausible yet nonexistent in the CDT taxonomy. These fabricated codes (e.g., “CDT-K0409” or “CDT-P0310”) typically resemble valid CDT identifiers, suggesting over-generalization of alphanumeric patterns. Errors are primarily *hallucinations* rather than omissions, though multi-code cases occasionally produce surplus restorative or crown-related codes not supported by the narrative.

#### 4.5.3. PEFT (Phi-1.5) Model

The PEFT model shows strong multi-label consistency but exhibits systematic over-generation in ambiguous cases. Similar to QLoRA, it sometimes fabricates CDT-like codes, but less frequently. The model’s primary weakness is the tendency to add extra restorative or endodontic procedures when multiple dental conditions coexist in the note. While hallucinations dominate, the model rarely omits required codes, indicating high sensitivity but lower precision.

#### 4.5.4. Bi-GRU Classifier

In contrast to LLM-based systems, the Bi-GRU model rarely hallucinates nonexistent or irrelevant CDT codes. Instead, its errors overwhelmingly involve *omissions*, especially in multi-procedure scenarios such as combined scaling, endodontics, and crown placement. Because the Bi-GRU depends on token-level sequence features without hierarchical clinical reasoning, it fails to capture secondary procedures implicitly tied to underlying conditions. While conservative and precise, it lacks the recall of LLM-based models in complex cases.

#### 4.5.5. Cross-Model Observations

LLM-based models tend to hallucinate additional codes due to their generative nature and exposure to both procedural and diagnostic terms. In contrast, the Bi-GRU model acts as a high-precision, low-recall classifier. Across all models, multi-procedure encounters present the greatest challenge, magnifying both hallucination (LLMs) and omission (Bi-GRU) patterns. These complementary strengths and weaknesses suggest that hybrid or ensemble strategies—combining LLM reasoning with classifier-level constraint filtering—may provide optimal performance for SNODENT-CDT coding.

#### 4.5.6. Detailed Breakdown of Error Types

To better understand the behavior of each model, we further categorized the observed errors into several common types. These categories help clarify how different modeling approaches fail under specific clinical scenarios.

##### Hallucinated Procedure Codes

**Figure 1:**
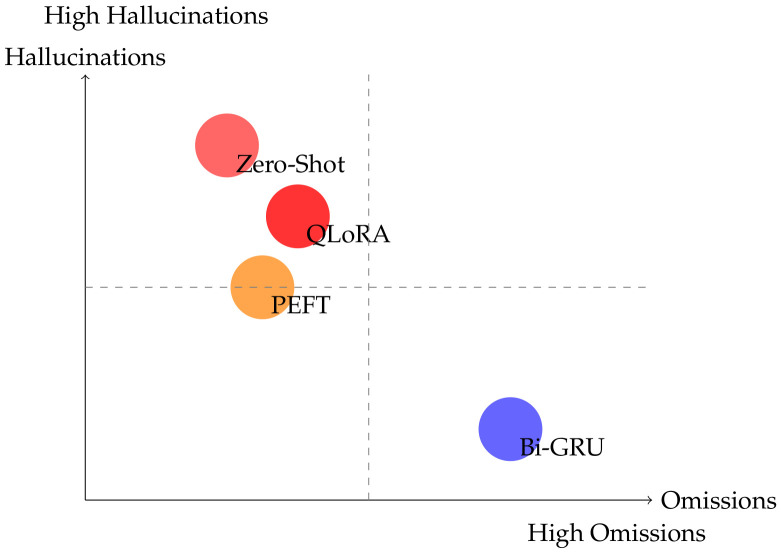
Hallucination vs. omission tendencies across models. LLM-based models cluster toward high hallucination rates, while Bi-GRU shows high omission rates, illustrating complementary strengths and weaknesses.

The most common error observed in LLM-based models was the generation of hallucinated procedure codes. These errors occur when the model predicts additional CDT codes that are not supported by the clinical note. In some cases, the models generated codes that resemble valid CDT identifiers but do not exist in the official CDT taxonomy. This behavior reflects the generative nature of LLMs, which may extrapolate plausible-looking codes based on learned alphanumeric patterns. Hallucination errors were particularly common in the QLoRA model, which produced a larger number of unique predicted labels compared to other approaches (Figure 1).

##### Diagnostic–Procedural Confusion

Another frequent error pattern involved confusion between diagnostic terminology and procedural codes. For example, the zero-shot LLM occasionally predicted SNODENT diagnostic identifiers (e.g., periodontal disease or pulpitis) as if they were CDT procedure codes. This occurs because the model associates diagnostic descriptions in the clinical narrative with procedural actions, even when a specific procedure is not explicitly documented.

##### Over-Prediction in Multi-Procedure Encounters

In cases where multiple dental procedures were described in a single note, LLM-based models sometimes predicted additional related procedures beyond those explicitly documented. For example, a note describing root canal therapy might also trigger predictions of crown placement or restorative procedures. These over-prediction errors suggest that LLMs sometimes infer downstream clinical interventions that are plausible but not explicitly documented in the encounter.

##### Procedure Omission

In contrast to the generative models, the Bi-GRU classifier most commonly exhibited omission errors. These occurred when the model failed to predict one or more valid CDT codes present in the gold standard annotation. Omission errors were particularly noticeable in notes describing multiple simultaneous procedures. Because the Bi-GRU relies on token-level sequence features without broader clinical reasoning, it may miss procedures that are implied by context rather than directly stated.

##### Comparative Error Behavior Across Models

Overall, the error patterns reveal a clear distinction between generative and discriminative approaches (Figure 1). LLM-based models tend to produce hallucinated or extra codes due to their open-ended generation process, while the Bi-GRU classifier tends to under-predict procedures due to its conservative classification behavior. These complementary error patterns suggest that hybrid systems combining LLM reasoning with classifier-based filtering or rule-based validation may provide improved accuracy for automated dental coding.

## 5. Discussion

This study presents one of the first comprehensive evaluations of Large Language Models (LLMs) and traditional deep learning methods for automated CDT dental procedure coding from free-text clinical notes. The findings highlight both the promise and the challenges of applying modern NLP approaches in an underexplored domain such as dental informatics. Across all experiments, three major themes emerged: (1) the surprising strength of zero-shot LLM performance on common CDT codes, (2) the limitations and trade-offs introduced by parameter-efficient fine-tuning, especially under quantization, and (3) the robust and balanced performance of traditional supervised models such as the Bi–GRU when sufficient labeled data are available. Together, these observations underscore the need for hybrid modeling strategies and domain-focused data resources to advance automated dental coding systems.

### 5.1. Effectiveness and Limitations of Zero-Shot LLM Reasoning

The zero-shot LLM (GPT-4o-mini) demonstrated exceptionally strong performance on high-frequency CDT codes, achieving the highest micro-F1 score (0.9614) and perfect micro-recall. This suggests that modern instruction-tuned models possess sufficient general clinical knowledge and semantic reasoning ability to infer dental procedures even without domain-specific training. Clinically, this is significant: it indicates that dental practices may benefit from out-of-the-box coding support even before investing in fine-tuning workflows.

However, the zero-shot model struggled with rare CDT codes, as reflected in the lower macro-F1 score (0.4543). The error analysis further revealed a recurring pattern of hallucinated additions, often involving SNODENT diagnostic terms misclassified as CDT procedures. These errors stem from the inherent generative nature of LLMs and their exposure to mixed taxonomies during pretraining. Although this behavior has minimal impact in single-procedure encounters, it becomes problematic in multi-procedure visits where accurate billing requires precise code boundaries. Therefore, while zero-shot prompting offers strong initial performance, its reliability depends on mechanisms for filtering hallucinated codes or constraining predictions via schema-based verification.

### 5.2. Impact of Fine-Tuning: Gains and Trade-Offs

Fine-tuning produced mixed results. The PEFT LoRA model outperformed QLoRA across all evaluation metrics, confirming that aggressive quantization introduces a meaningful performance penalty in small-domain datasets. The QLoRA model exhibited substantially higher Hamming loss and a tendency to hallucinate realistic-but-invalid CDT codes (e.g., fabricated alphanumeric sequences resembling true CDT identifiers). These behaviors reflect well-known challenges of applying low-bit quantization to tasks that require precise, schema-constrained outputs.

In contrast, the non-quantized PEFT LoRA model achieved substantially higher exact-match accuracy (0.79) and improved handling of rare codes, suggesting that fine-tuning—when performed without compression—helps models internalize domain-specific terminology and multi-label structures. This supports the broader observation that full-precision or lightly compressed adapters are better suited for specialized clinical tasks involving small ontologies and structured outputs. Fine-tuning also reduced the frequency of SNODENT-to-CDT confusion observed in the zero-shot model, indicating that labeled examples help LLMs learn to separate diagnostic and procedural vocabularies.

Nonetheless, fine-tuned LLMs still produced more hallucinations than the Bi–GRU model, and their gains were limited by the small dataset size. The results emphasize the importance of high-quality, diverse, and sufficiently large training corpora to maximize the benefits of fine-tuning in dental coding applications.

### 5.3. Strengths and Constraints of Traditional Deep Learning Models

The Bi–GRU exhibited the most balanced performance profile, with nearly identical micro- and macro-F1 scores (0.9362 and 0.9377). This indicates strong and consistent performance across both common and rare CDT codes, a property not matched by any LLM-based approach evaluated in this study. The Bi–GRU’s low Hamming loss and clean error patterns highlight its suitability for structured multi-label classification when labeled training data are available.

However, the Bi–GRU lacks the contextual reasoning abilities of LLMs and occasionally omitted valid CDT codes in multi-procedure encounters—an expected limitation given its fixed-length token-based representation and lack of instruction-following capacity. Unlike LLMs, the Bi–GRU cannot infer implicit procedures implied by narrative context (e.g., a fractured cusp suggesting crown placement) unless such patterns are explicitly learned from the training data. Thus, while the Bi–GRU offers reliability and precision, its performance is constrained by dataset coverage and the absence of cross-sentence clinical reasoning.

### 5.4. Error Patterns Reveal Complementary Strengths

The comparative error analysis reveals that LLMs and traditional models make fundamentally different errors. LLMs tend to hallucinate additional CDT codes, especially in ambiguous or multi-procedure visits, while the Bi–GRU tends to under-predict, omitting codes that require contextual inference. These complementary tendencies suggest that hybrid or ensemble frameworks—combining LLM reasoning with classifier-based filtering or rule-based validation—may provide superior accuracy and robustness. For example, an LLM could propose a set of candidate CDT codes, and a discriminative model like the Bi–GRU could serve as a precision gate to validate or reject improbable predictions. Such hybrid strategies have shown promise in medical coding research and may be particularly effective in the dental domain.

### 5.5. Comparison with Previously Published Studies

The performance patterns observed in this study align with, but also meaningfully diverge from, previously published work in both medical coding and dental NLP. In the broader medical domain, automated ICD coding systems have demonstrated strong micro-F1 performance using deep learning architectures. For example, Mullenbach et al. reported micro-F1 scores approaching 0.54–0.63 on large-scale ICD-9 prediction tasks using attention-based CNN architectures trained on tens of thousands of discharge summaries [15]. Similarly, Xu et al. demonstrated that recurrent and hybrid neural models can achieve competitive performance in multi-label medical code assignment tasks when trained on large, curated datasets [13]. Compared to these benchmarks, the micro-F1 scores observed in our study (0.9136–0.9614 for LLM-based models and 0.9362 for Bi–GRU) appear substantially higher. However, this difference must be interpreted in context: medical ICD coding involves thousands of possible codes and highly heterogeneous documentation, whereas CDT coding encompasses a smaller, more structured taxonomy. Furthermore, our dataset is synthetic and controlled, which likely reduces linguistic ambiguity relative to real-world EHR corpora.

Within the dental domain, prior NLP studies have primarily focused on diagnostic entity extraction rather than procedural billing. Keels et al. reported weighted F1 scores exceeding 0.97 for periodontal diagnosis extraction using a fine-tuned RoBERTa model trained on synthetic dental data [10]. Our Bi–GRU macro-F1 score of 0.9377 and LLM micro-F1 values above 0.90 fall within a comparable range of performance, suggesting that modern neural models can achieve high structured prediction accuracy in dental clinical text when the label space is constrained. However, the tasks differ fundamentally. Periodontal diagnosis extraction is closer to named entity recognition or single-label classification, whereas CDT coding is a multi-label procedural billing task requiring simultaneous identification of multiple co-occurring interventions within a single encounter. Multi-label procedure coding introduces additional complexity, particularly in distinguishing primary and secondary procedures and preventing over-generation.

Recent work by Chuang et al. demonstrated strong performance in dental entity extraction workflows that leverage large language models for prompt generation combined with downstream transformer-based classifiers [17,18]. Their findings emphasize the value of LLM-assisted pipelines and highlight cross-institutional generalization challenges. Our results extend this paradigm by directly evaluating LLMs as inference engines for structured CDT code generation, rather than solely as data augmentation or annotation tools. Notably, our analysis reveals a recurring hallucination tendency in LLM-based models—a behavior less prominent in entity extraction tasks but increasingly reported in generative coding systems. This generative over-prediction contrasts with the conservative omission pattern observed in the Bi–GRU classifier, underscoring architectural differences between generative and discriminative modeling approaches.

Another key distinction between our findings and the prior literature concerns the divergence between micro- and macro-level performance metrics. In many medical coding studies, macro-F1 scores tend to be substantially lower than micro-F1 due to extreme label imbalance [15]. We observe a similar but more moderate phenomenon in LLM-based CDT coding, where strong micro-F1 performance on frequent procedures contrasts with weaker macro-F1 results for rare codes. The Bi–GRU model, in contrast, demonstrates near parity between micro- and macro-F1, suggesting more uniform learning across the limited CDT label space. This difference may reflect the advantage of fully supervised discriminative models when trained on balanced or moderately sized taxonomies.

Taken together, these comparisons suggest that while CDT coding shares methodological similarities with medical ICD coding and dental entity extraction, it represents a distinct structured multi-label generation task with unique challenges. Our findings contribute to the growing body of dental NLP literature by demonstrating that modern LLM-based approaches can achieve high micro-level performance in procedural coding, but require careful fine-tuning and validation to manage hallucinations and ensure robustness across the full CDT taxonomy.

### 5.6. Implications for Real-World Deployment in Dental Informatics

From a clinical workflow perspective, automated CDT coding systems can substantially reduce administrative burden, decrease claim denials, and promote consistency in billing practices. The results indicate that even zero-shot LLM-based systems may be useful for rapid prototyping or real-time coding assistance in small dental practices. However, reliable deployment at scale—especially in environments with diverse documentation styles—will require careful fine-tuning and post-processing safeguards to mitigate hallucinations.

The strong performance of the Bi–GRU demonstrates that traditional models remain highly valuable in settings where labeled training data are available. This suggests a practical pathway for dental organizations seeking to build customized coding tools: leverage LLMs for bootstrapped dataset creation, followed by supervised training of smaller models optimized for operational use.

We acknowledge that many contemporary electronic dental record (EDR) systems provide structured, template-based documentation workflows in which predefined procedure texts are directly linked to corresponding CDT billing codes. In such environments, clinicians can select a predefined template for common procedures (e.g., prophylaxis, extraction, crown placement), modify tooth numbers or procedural details, and generate billing codes automatically without requiring post hoc NLP-based extraction. This structured workflow can be efficient and reduces reliance on narrative interpretation.

However, several practical scenarios motivate continued investigation of AI-driven coding systems. First, not all clinical environments consistently use standardized templates; free-text documentation remains common in multi-provider practices, legacy systems, academic settings, and during documentation of complex or atypical encounters. Second, template misuse or incomplete template selection can lead to mismatches between documented procedures and assigned billing codes, where AI-based validation could serve as an auditing or safety layer. Third, AI-driven systems may provide value in retrospective chart review, insurance audit preparation, revenue cycle optimization, and quality assurance, where automated reconciliation between narrative documentation and coded claims is required. Finally, even in structured workflows, AI models may support hybrid approaches by suggesting additional or missing procedures based on contextual clinical descriptions.

Therefore, rather than replacing structured template-based documentation, AI-driven CDT extraction should be viewed as a complementary tool that can enhance coding validation, detect discrepancies, and support environments where documentation practices are heterogeneous. The present study focuses on technical feasibility and model behavior under controlled conditions, with real-world workflow integration representing an important direction for future investigation.

### 5.7. Future Directions

This study highlights several opportunities for future research:

- Expanding labeled datasets: Larger, real-world dental corpora will be essential for improving fine-tuning fidelity and addressing rare CDT codes.
- Hybrid modeling strategies: Combining LLM proposal generation with discriminative filtering may mitigate hallucinations while retaining deep contextual reasoning.
- Schema-constrained decoding: Developing constrained generation methods that enforce CDT taxonomy validity could substantially reduce hallucinated outputs.
- Integration with SNODENT ontologies: Leveraging the structure of SNODENT may help distinguish diagnostic language from procedural descriptions.
- Real-time clinical evaluation: Future work should include usability studies and workflow integration trials within EDR systems.

Overall, this study demonstrates that both LLMs and traditional neural models hold significant promise for automating CDT coding, with each offering distinct advantages. Continued development of domain-specific datasets, fine-tuning strategies, and hybrid architectures will be critical for realizing reliable, scalable, and clinically aligned dental coding solutions.

## 6. Limitations

While this work provides a robust foundation for automated CDT dental coding, several limitations should be acknowledged. First, the dataset used in this study is synthetic and, although carefully designed to mimic real-world clinical documentation, cannot fully capture the linguistic diversity, variability in provider writing styles, and idiosyncratic documentation habits found in operational dental practices. Performance on real clinical notes may differ, particularly with respect to ambiguous descriptions, inconsistent terminology, and incomplete documentation. The controlled and partially templated nature of the synthetic notes may have reduced ambiguity and increased lexical consistency, potentially inflating performance metrics relative to what would be observed in authentic clinical environments.

Accordingly, the results presented here should be interpreted as a proof-of-concept demonstration of methodological feasibility rather than evidence of deployment readiness. External validation on de-identified real clinical notes is a critical next step for assessing ecological validity, robustness, and generalizability of the proposed framework.

Second, the fine-tuning experiments were conducted on a relatively small dataset, which may limit the generalizability of learned patterns and hinder the models’ ability to robustly identify rare CDT codes. The QLoRA model, in particular, showed sensitivity to low-bit quantization, suggesting that larger or more diverse datasets may be necessary to stabilize parameter-efficient fine-tuning in the dental domain.

A third limitation relates to hallucination and omission patterns observed in the models. LLM-based approaches, especially in their zero-shot and fine-tuned forms, exhibited a tendency to hallucinate additional CDT codes or fabricate codes that do not exist in the CDT taxonomy. These behaviors pose potential risks for real-world deployment, where incorrect billing codes may have financial or regulatory consequences. Conversely, the Bi–GRU model occasionally omitted valid procedures, particularly when contextual reasoning was required. Developing schema-constrained decoding and validation mechanisms will be essential for mitigating these risks.

Finally, this study did not evaluate the usability, workflow integration, or real-time performance of the system within dental practices or Electronic Dental Record (EDR) environments. Operational considerations such as latency, model interpretability, clinician trust, and integration with existing billing workflows remain important directions for future investigation.

Despite these limitations, the present work offers meaningful insights into the strengths and weaknesses of state-of-the-art NLP approaches for dental coding and highlights clear opportunities for advancing the field through expanded datasets, improved modeling techniques, and real-world evaluation.

### Strategies to Mitigate Current Study Limitations

While this study demonstrates promising results for automated CDT coding, several steps can be taken to reduce the identified limitations and strengthen future iterations of this work.

First, the use of a synthetic dataset, although valuable for controlled experimentation and reproducibility, limits direct generalizability to real-world clinical environments. To mitigate this limitation, future work will involve validation on de-identified electronic dental record (EDR) datasets obtained under appropriate institutional review board (IRB) approval. Evaluating model performance on authentic provider-authored documentation—including abbreviations, incomplete charting, and stylistic variability—will provide a more realistic assessment of deployment readiness.

Second, expanding dataset size and diversity will improve model robustness, particularly for rare CDT codes. Collecting multi-institutional corpora and incorporating diverse provider writing styles can reduce overfitting to templated narrative structures and enhance macro-level performance. Data augmentation strategies and curriculum-based training may further improve rare-label learning.

Third, hallucination behaviors observed in LLM-based models can be mitigated through schema-constrained decoding, taxonomy-aware filtering, and post-processing validation against the official CDT code list. Implementing constrained generation methods that restrict outputs to valid CDT identifiers would reduce fabricated codes and improve billing safety.

Fourth, hybrid modeling approaches may help balance hallucination and omission patterns. For example, an LLM could generate candidate CDT codes, followed by a discriminative classifier or rule-based validator that filters improbable or unsupported predictions. Such ensemble strategies may leverage the contextual reasoning strengths of LLMs while preserving the precision of supervised classifiers.

Fifth, we acknowledge that multi-label CDT classification may involve class imbalance, and that k-fold or stratified multi-label cross-validation would provide more robust generalization estimates.

Calibration analysis and threshold optimization for multi-label predictions may further enhance deployment reliability. All models were evaluated using the same fixed train–test split to ensure direct comparability. provide more robust generalization estimates.

Finally, prospective workflow evaluation studies—including clinician-in-the-loop assessments—are necessary to determine usability, trust, latency, and real-world billing impact. Integrating model predictions into EDR systems and measuring reductions in administrative burden or claim denial rates would provide meaningful operational validation.

Collectively, these steps offer a clear roadmap for addressing current limitations and advancing automated dental coding systems toward safe, scalable clinical deployment.

## 7. Conclusions

This study evaluated multiple artificial intelligence approaches for automated CDT dental procedure coding from free-text clinical notes. The main findings are summarized as follows:

1. Zero-shot LLM performance: The zero-shot LLM achieved the highest micro-F1 score (0.9614) and perfect recall for frequent CDT codes, demonstrating strong contextual reasoning without task-specific training.
2. Impact of fine-tuning: Parameter-efficient fine-tuning improved domain alignment, with non-quantized LoRA outperforming QLoRA across all evaluation metrics. However, fine-tuned LLMs exhibited over-generation tendencies in some multi-label cases.
3. Bi–GRU robustness: The supervised Bi–GRU model achieved balanced performance across both micro- and macro-level metrics (micro-F1 = 0.9362; macro-F1 = 0.9377), with minimal hallucinated outputs but occasional omissions in complex encounters.
4. Generative vs. discriminative trade-offs: LLM-based approaches tended toward hallucination, whereas the Bi–GRU model demonstrated conservative prediction behavior, highlighting complementary strengths between generative and supervised architectures.
5. Feasibility of automated CDT coding: The results collectively demonstrate that multi-label CDT extraction from dental clinical text is technically feasible using modern NLP methods, with performance levels suitable for further validation and refinement.

Overall, this work establishes a comparative technical foundation for AI-driven dental procedure coding and provides empirical insights into the strengths and limitations of LLM-based and traditional neural approaches.

## Figures and Tables

**Table 1:** Comparison of modeling approaches for CDT dental code prediction, including training requirements, inference workflows, and evaluation metrics.

Approach	Training	Train/Test Split	How It Works (Inference)	Metrics Used
Low/Zero-Shot (GPT-4o-mini)	No training (prompt-based)	None	Prompt + few-shot examples → API call → extract JSON → parse CDT codes	micro/macro F1, precision, recall, exact match, Hamming loss
QLoRA (TinyLlama 1.1B)	Yes (LoRA on 4-bit model)	90% train/10% test	Fine-tuned using QLoRA → HuggingFace generation pipeline → JSON parsing	Same as above
PEFT (LoRA)	Yes (LoRA, no 4-bit quantization)	90% train/10% test	Fine-tuned model → pipeline generation → JSON parsing	Same as above
Bi-GRU	Yes (traditional neural network)	80% train/20% test (10% validation)	Tokenize text → Bi-GRU classifier → sigmoid outputs thresholded at 0.5	micro/macro F1, Hamming loss, per-label classification report

**Table 2:** Fine-tuning hyperparameter configuration for QLoRA and LoRA models.

Parameter	QLoRA Configuration	LoRA Configuration
Base Model	TinyLlama-1.1B-Chat-v1.0	Phi-1.5
Quantization	4-bit NF4	None
Compute dtype	bfloat16	FP16
Double Quantization	Enabled	Not used
LoRA Rank (*r*)	64	8
LoRA Alpha (α)	16	16
LoRA Dropout	0.05	0.05
Target Modules	qkv_proj, o_proj	q_proj, v_proj, o_proj
Training Epochs	1	1
Learning Rate	$2\times 10^{-4}$	$2\times 10^{-4}$
Per-device Batch Size	2	1
Gradient Accumulation Steps	4	4
Effective Batch Size	8	4
Maximum Token Length	256	256
Train/Test Split	90%/10%	90%/10%
Random Seed	42	42

**Table 3:** Distribution of CDT Codes in the Dataset.

CDT Code	Procedure Description	Frequency
CDT-D4341	Periodontal scaling and root planing (4+ teeth per quadrant)	3530
CDT-D2740	Crown—porcelain/ceramic substrate	2213
CDT-D1110	Adult prophylaxis	1938
CDT-D7140	Extraction of erupted tooth or exposed root	1492
CDT-D3330	Endodontic therapy—molar root canal	1216
CDT-D3320	Endodontic therapy—bicuspid root canal	1181
CDT-D3310	Endodontic therapy—anterior root canal	1179
CDT-D2392	Resin-based composite restoration, two surfaces	1064
CDT-D2140	Amalgam restoration, one surface	984
CDT-D6010	Surgical placement of implant body	71
Total Unique CDT Codes		10

**Table 4:** Comparison of model performance across Low/Zero-Shot prompting, QLoRA fine-tuning, and PEFT LoRA fine-tuning.

Metric	Low/Zero-Shot	QLoRA	PEFT (LoRA)
micro_f1	0.9614	0.7831	0.9136
macro_f1	0.4543	0.2165	0.3614
micro_precision	0.9257	0.6435	0.8409
macro_precision	0.4540	0.2033	0.3540
micro_recall	1.0000	1.0000	1.0000
macro_recall	0.4545	0.2381	0.3704
exact_match	0.8912	0.3900	0.7900
hamming_loss	0.0054	0.0195	0.0104
num_labels	22	42	27

**Table 5:** Performance of the Bi-GRU neural network on CDT code prediction.

Metric	Bi-GRU Results
micro_f1	0.9362
macro_f1	0.9377
hamming_loss	0.0185

## Data Availability

The original contributions presented in this study are included in the article and Supplementary Material. Further inquiries can be directed to the corresponding author.

## Supplementary Materials

The following supporting information can be downloaded at: https://www.mdpi.com/article/10.3390/dj14060339/s1.
